# An Elusive Case of Acute Interstitial Pneumonia

**DOI:** 10.7759/cureus.4685

**Published:** 2019-05-17

**Authors:** Jennifer Obasi

**Affiliations:** 1 Internal Medicine, Medical College of Wisconsin Affiliated Hospitals, Milwaukee, USA

**Keywords:** hypoxia, acute interstitial pneumonia, hamman-rich syndrome, cough, aip, dyspnea

## Abstract

Acute interstitial pneumonia (AIP) is a rare and severe form of diffuse lung injury that affects previously healthy individuals. It is a subset of idiopathic lung disease with a rapidly progressive course. The prognosis is poor with over 50% of patients dying during hospital admission from progressive respiratory failure. Presented herein is a case of a previously healthy 64-year-old woman without a prior history of tobacco use who presented with progressive hypoxia, dyspnea, and nonspecific constitutional symptoms.

## Introduction

Acute interstitial pneumonia (AIP) is a rare and often severe subset of idiopathic lung disease with a rapidly progressive course. It typically affects otherwise healthy individuals and presents with acute to subacute progressive hypoxic respiratory failure. It occurs with equal frequency in men and women with a mean age of onset of 50 to 55 years [1]. AIP manifests the histopathologic appearance of diffuse alveolar damage (DAD) [2] which can occur in a myriad of other known causes of lung injury, including acute respiratory distress syndrome (ARDS), connective tissue diseases, drugs, and acute hypersensitivity pneumonitis. In the case of AIP, it is idiopathic. High-resolution computed tomography (HRCT) is more sensitive than plain chest radiographs for the diagnosis of AIP with areas of nonspecific patchy bilateral consolidation and ground-glass opacities seen on imaging [2-3]. In the right clinical context, the diagnosis is supported by the acute onset of respiratory failure with bilateral lung infiltrates on imaging in the absence of an identifiable cause or predisposing condition. A lung biopsy is often necessary to confirm the diagnosis [3]. Histopathologic changes seen on biopsy include thickening of the alveolar septa due to interstitial edema, inflammatory cell infiltrates, hyaline membranes, and thrombi in small pulmonary arteries [3]. Other known causes of DAD and other causes of diffuse pulmonary opacities, including infectious processes and cardiogenic pulmonary edema, must be excluded [3]. Treatment is supportive, with most patients requiring mechanical ventilation. Glucocorticoids are usually given, although efficacy is unclear. Alternative immunosuppressive therapies have been reported in published case reports, although success has been limited.

## Case presentation

**Figure 1:**
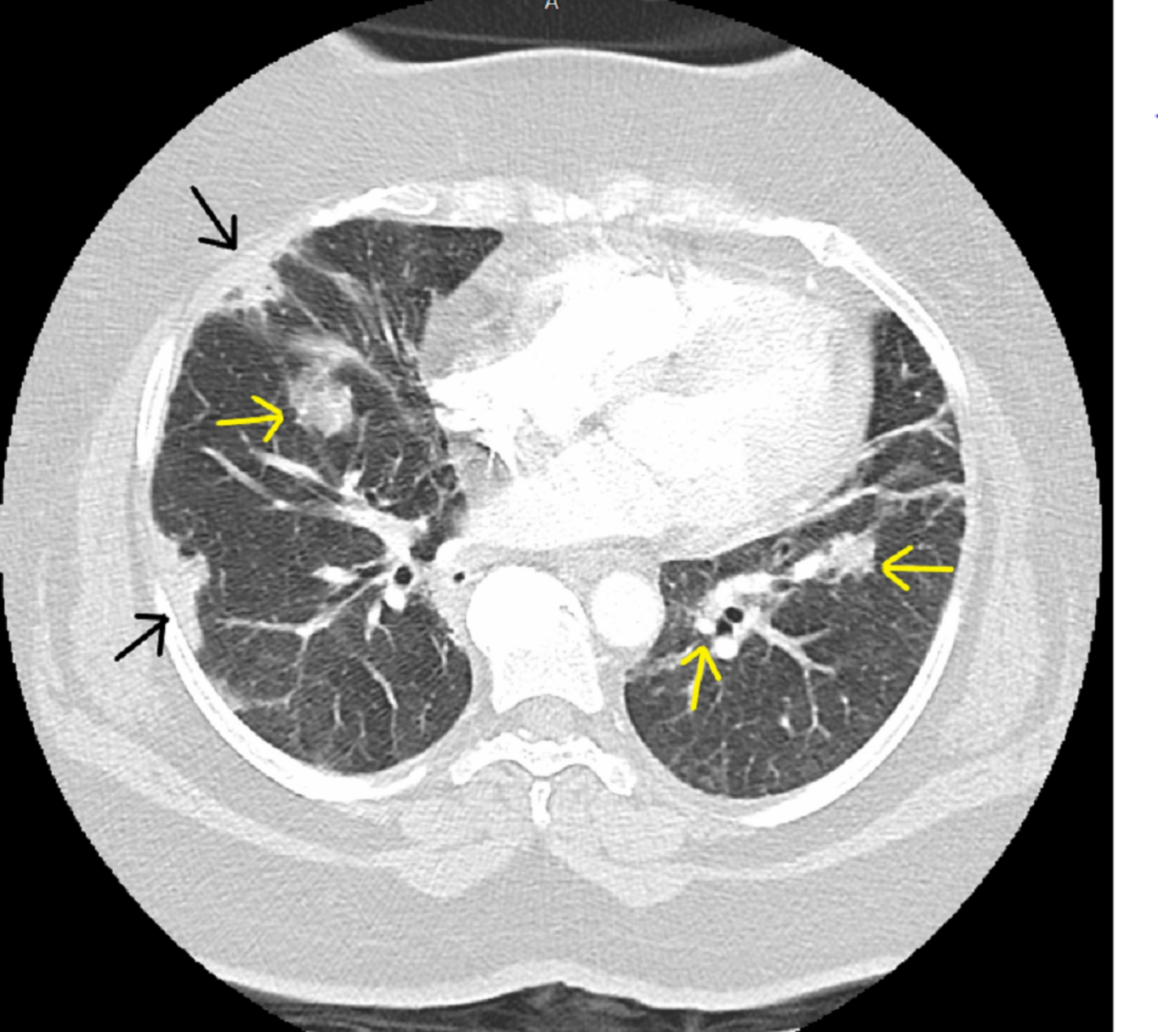
Scattered patchy infiltrative changes involving the periphery (yellow arrows), the anterolateral segment of the right upper lobe, and the anterior and lateral segments of the left lower lobe (black arrows). No focal dominant masses were noted.

A previously healthy 64-year-old woman with a history of hypertension who presented with arthralgias, myalgias, subjective fevers, and a dry cough with progressive dyspnea for two weeks was admitted to an outside hospital (OSH) for further evaluation. Her workup included negative studies for respiratory cultures, respiratory viral and atypical pneumonia pathogens, blood cultures, urine Streptococcus, Legionella, Histoplasma, and Blastomyces antigens. Other workup included a complete blood count which demonstrated mild normocytic anemia (hemoglobin 10.2 - 11.1 g/dL; prior baseline 13 - 14 g/dL) with normal white blood cell count and modest elevations in erythrocyte sedimentation rate (66 mm/hr) and C reactive protein (1.1 mg/dL). Scl-70, rheumatoid factor, anticentromere, cytoplasmic antineutrophil cytoplasmic antibodies (C-ANCA), and perinuclear antineutrophil cytoplasmic antibodies (P-ANCA) antibody titers were within normal parameters. Additional workup revealed elevated cyclic citrullinated peptide (CCP) antibodies IgG/IgA to 78. CT of the chest was negative for pulmonary emboli but revealed multiple scattered patchy infiltrative changes thought to represent bronchopneumonia (Figure 1).

**Figure 2:**
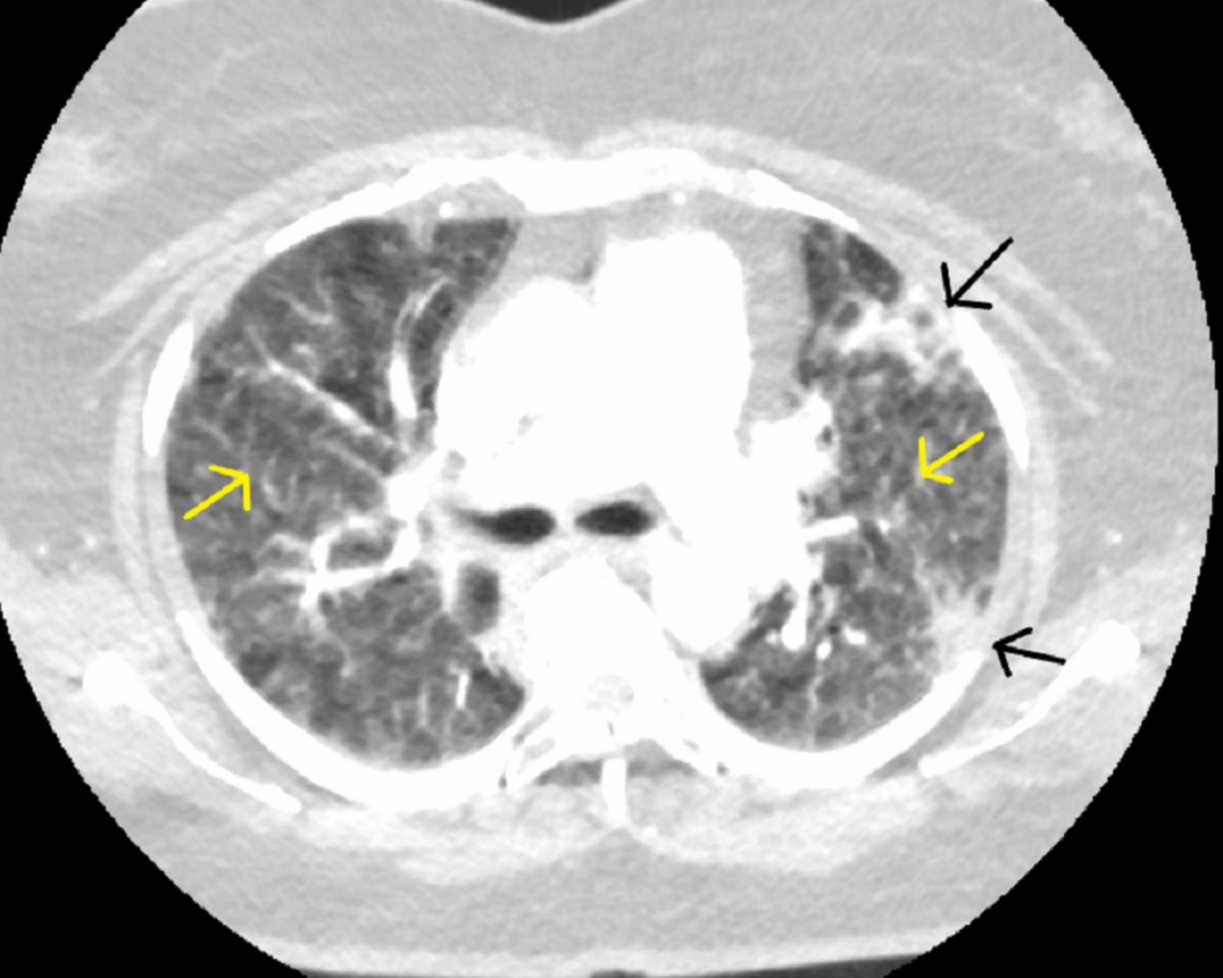
Study compromised by respiratory motion; however, no main or segmental pulmonary artery embolus identified. Subpleural consolidations present (black arrows) with mosaic attenuation (yellow arrows).

Despite broad-spectrum antimicrobial treatment, her respiratory status continued to worsen and she self-discharged after eight days at the OSH and presented to our institution to seek a “second opinion.” Physical exam revealed mild hypoxia and tachycardia with inspiratory crackles in the upper lung zones. Repeat CT scan showed extensive subpleural consolidations throughout all pulmonary lobes, extensive mosaic attenuation with some interstitial thickening, and no main or segmental artery pulmonary embolus identified (Figure 2).

**Figure 3:**
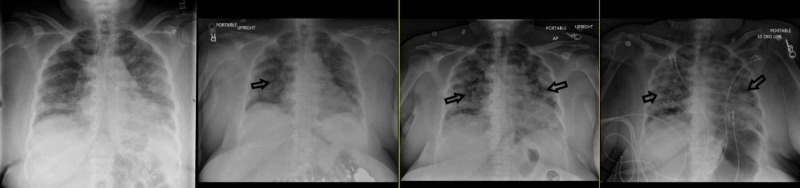
Chest x-ray progression, left to right, showing worsening diffuse bilateral patchy opacities (black arrows) with progressive obscuration of the cardiac silhouette.

Posteroanterior and portable chest x-rays (CXR) were obtained with serial imaging showing worsening bilateral opacities (Figure 3).

A transthoracic echocardiogram (TTE) was done to exclude any cardiac etiology and was unremarkable. Additionally, pulmonary artery systolic pressure and right ventricular function on the TTE were normal. Given the elevation in CCP antibodies, empiric high-dose steroids were started for possible rheumatoid lung disease. She was subsequently transferred to the intensive care unit (ICU) where she was intubated, mechanically ventilated, and bronchoscopy with bronchoalveolar lavage (BAL) was completed. She continued to experience refractory hypoxia after the bronchoscopy, and the decision was made by the family to withdraw care 12 days later. BAL analysis was negative for infectious etiologies. An autopsy completed on the lung tissue showed diffuse alveolar damage with widespread hyaline membrane formation in alveolar spaces and multiple bilateral pulmonary emboli. 

## Discussion

As previously stated, AIP typically occurs in previously healthy individuals, in their fifth decade of life, and without any prior history of lung disease, although the patient in this case presented in her sixth decade of life. Like this patient, the presented history is usually negative for tobacco use. Clinically, it is characterized by nonspecific viral-like symptoms with fever, cough, and dyspnea, followed by acute hypoxic respiratory failure with bilateral opacities on imaging and lack of an identifiable etiology or predisposing condition [3]. Clubbing is typically not seen.

DAD is characterized by thickening of the alveolar septa due to interstitial edema, inflammatory cell infiltration, hyaline membranes in focal areas along the alveolar septa, and thrombi in small pulmonary arteries [3]. The patient’s autopsy findings supported this diagnosis. In evaluating patients who present with suspected AIP, it is imperative to exclude cardiogenic pulmonary edema and other known causes of diffuse pulmonary opacities, including connective tissue diseases such as rheumatoid arthritis, primary Sjogren syndrome, systemic lupus erythematosus, systemic sclerosis, polymyositis, and dermatomyositis. The patient above did have elevated anti-CCP antibodies, but her clinical picture otherwise did not fit with rheumatoid arthritis or rheumatoid lung disease. A prior study characterized a cohort of patients with lung disease and a history of tobacco use, anti-CCP positivity, and without rheumatoid arthritis or other connective tissue diseases [4]. Treatment is primarily supportive with supplemental oxygenation and mechanical ventilation. Most patients also receive a trial of corticosteroids, but the prognosis remains poor [3]. In addition to mechanical ventilation, our patient also received treatment with broad-spectrum antibiotics and high-dose steroids, but despite this, her condition continued to worsen.

## Conclusions

AIP is a rare form of diffuse lung injury that affects previously healthy individuals. Treatment is supportive and prognosis remains poor.
